# Viral metagenomics reveals sapoviruses of different genogroups in stool samples from children with acute gastroenteritis in Jiangsu, China

**DOI:** 10.1007/s00705-020-04549-y

**Published:** 2020-02-11

**Authors:** Wang Li, Surong Dong, Juan Xu, Xiaobin Zhou, Junling Han, Zhaqing Xie, Qin Gong, Hailin Peng, Chenglin Zhou, Mei Lin

**Affiliations:** 1 Taizhou People’s Hospital, 366 Taihu Road, Taizhou, 225300 Jiangsu China; 2 The Fifth Affiliated Hospital of Nantong University, Taizhou, 225300 Jiangsu China

## Abstract

Sapoviruses (SAVs), including several genogroups (GI to GV), are one of the causes of acute gastroenteritis (AGE). In this study, viral metagenomics revealed the presence of sapoviruses of different genogroups in stool from children with AGE. Eight different complete SAV genomes were determined, of which five belonged to GI and the other three belonged to GII, GIV and GV, respectively. Although they were highly similar to published sequences, the GIV and GV were the first complete genome sequences of these SAVs found in China. In a prevalence investigation, 19% of subjects with AGE were positive for SAVs, while none of the control group was positive.

Sapoviruses (SAVs) belong to the family of *Caliciviridae* and cause both sporadic cases and occasional outbreaks of acute gastroenteritis (AGE) in humans and animals worldwide. Although all ages are affected, children younger than five years have the highest burden of disease. Recently, studies have shown that infections with SAVs can result in hospitalization and severe dehydration [1–3]. SAVs have increasingly been detected in low- and middle-income countries [4], but unfortunately, no licensed vaccines or antivirals are available for SAV infections. SAVs are genetically diverse viruses that can be classified into several genogroups (GI to GV) [5], four of which (I, II, IV, and V) infect humans. The existence of several additional SAV genogroups infecting different animal species has been shown [6]. Although SAV is known to cause acute gastroenteritis worldwide, the genetic characteristics of human SAVs in China are not known.

In 2016, 100 fecal samples were collected from children (≤ 6 years old) with suspected viral AGE from the cities of Taizhou (n = 50), Zhenjiang (n = 30) and Xuzhou (n = 20) in Jiangsu province. Antigen testing at the local hospitals for viral pathogens, including norovirus, astrovirus, and rotavirus, gave negative results. As controls, 40 fecal samples (20 from Taizhou, 10 from Zhenjiang, and 10 from Xuzhou) were collected in 2016 from healthy children (≤ 6 years old) who had shown no symptoms of diarrhea within at least eight weeks before or after sample collection. Ethical approval was given by the Ethics Committee of Taizhou Hospital with the reference no. TZRMYY2015023.

To detect viral nucleic acids, 10% suspensions of fecal samples were prepared in phosphate-buffered saline and mixed thoroughly by vortexing. Fecal suspensions were then centrifuged for 10 min at 15,000 × *g*. One hundred μL of supernatant from each of the 140 samples was collected and combined into 14 pools based on sampling region and health status, each including 10 samples (Table 1). For viral metagenomic analysis, sample treatment, library construction, and data processing were done as described previously [7–9]. To investigate the sequence read distribution of different types of viruses detected in these pooled libraries, BLASTx results based on clean unassembled sequence reads from each pooled library were further analyzed in the MEGAN program, which yielded the viral read distribution in the 14 libraries.

**Table 1:** Information about the sample pools included in the viral metagenomic analysis

Pool ID	Sample size	Group	Sampling site
Dia01	10	AGE	Taizhou
Dia02	10	AGE	Taizhou
Dia03	10	AGE	Taizhou
Dia04	10	AGE	Taizhou
Dia05	10	AGE	Taizhou
Dia06	10	AGE	Zhenjiang
Dia07	10	AGE	Zhenjiang
Dia08	10	AGE	Zhenjiang
Dia09	10	AGE	Xuzhou
Dia10	10	AGE	Xuzhou
Norm1	10	Normal	Taizhou
Norm2	10	Normal	Taizhou
Norm3	10	Normal	Zhenjiang
Norm4	10	Normal	Xuzhou

As shown in Fig. 1A, the viral sequences detected in the 14 libraries were compared to those of members of the families *Caliciviridae*, *Picornaviridae*, *Anelloviridae*, *Picobirnaviridae*, *Parvoviridae*, *Reoviridae*, *Astroviridae*, and *Adenoviridae*. Viruses of the families *Caliciviridae*, *Reoviridae*, *Astroviridae* and *Adenoviridae* are generally associated with AGE. Figure 1A also shows the sequence reads related to SAVs, which have rarely been reported to be associated with AGE in China. We detected SAV sequences in eight out of the ten pooled libraries from the AGE group, belonging to genogroups GI, GII, GIV and GV, while only a few reads of GIII, which is non-pathogenic for humans, were detected in the control group. From the eight SAV-positive pooled libraries, eight different complete SAV genome sequences were obtained by de novo assembly of the SAV reads in the individual pooled library, combined conventional PCR, which was used for bridging sequence gaps. Of the eight SAV-positive pooled libraries, one (Dia03) yielded too few SAV sequence reads to allow a complete genome sequence to be obtained, another (Dia04) yielded two different complete genome sequences, belonging to GI and GV, and the remaining six yielded one complete genome sequence. Of the eight complete SAV genomes identified, five belonged to GI and the other three belonged to GII, GIV, and GV, respectively.

**Fig. 1:**
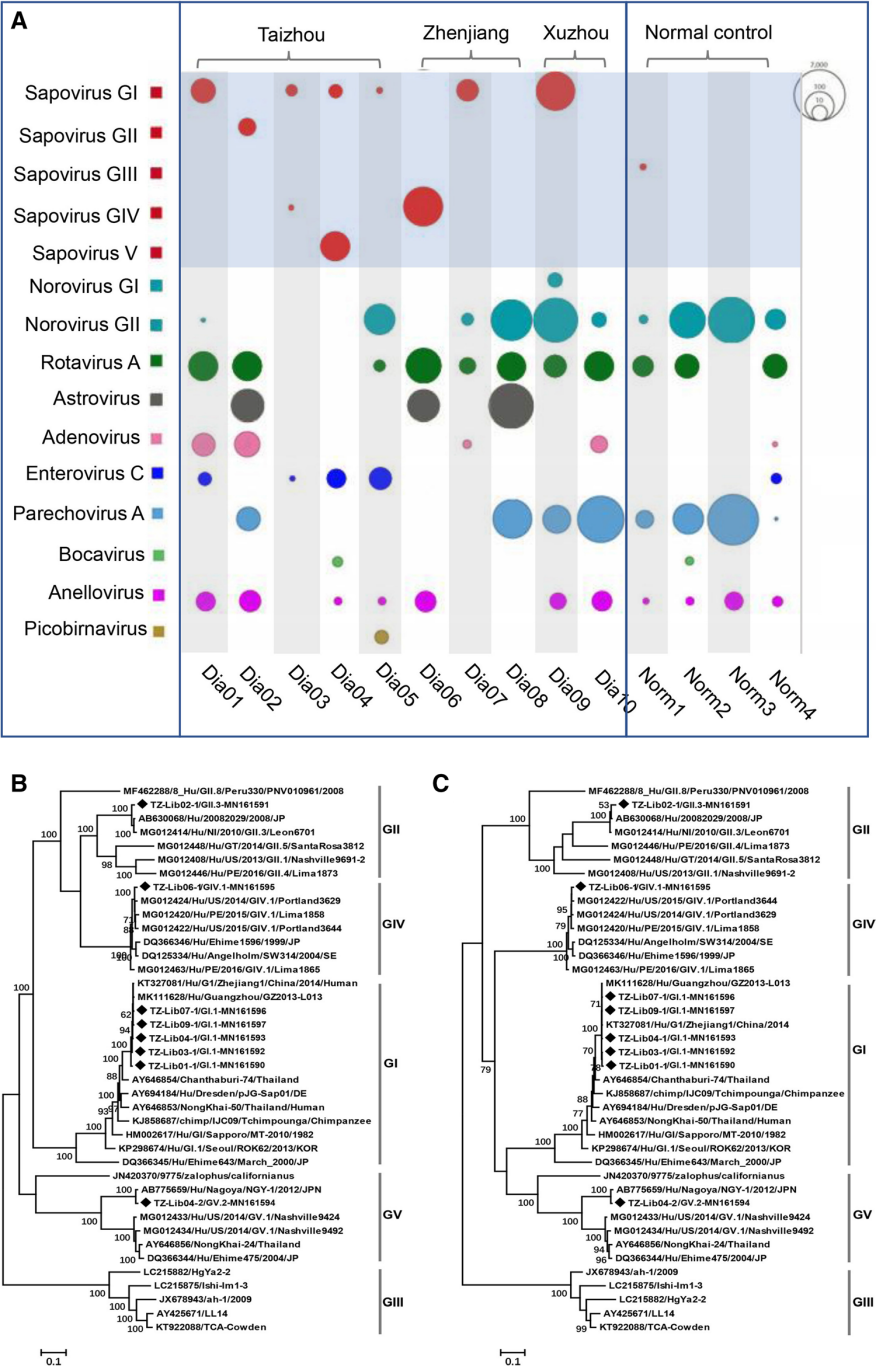
Virome composition and phylogeny. Virome composition in fecal samples from children with and without AGE. (**B**) Phylogenetic analysis based on the complete genome. (**C**) Phylogenetic analysis based on VP1 region

Maximum-likelihood phylogenetic trees were constructed based on the nucleotide sequences of the complete genome and the VP1 region using MEGA 7.0 with 1,000 bootstrap resamplings of the alignment data. The analysis included the eight complete genome sequences determined in this study, their best matches found in a BLASTn search, and complete genome sequences of representatives of each genogroup. Both trees indicated that the five SAV GI strains clustered closely with SAVs belonging to the GI.1 genotype. The remaining three strains clustered within GII.3, GIV.1, and GV.2, respectively, and were closely related to their best BLASTn matches (Fig. 1B and C). Based on their complete genome sequences, the five GI strains shared 98.2–98.9% sequence identity to two previous Chinese SAV strains, KT327081 and MK111628, which were isolated from patients in Zhejiang province and Guangdong province, respectively. The GII strain showed 97.9% sequence identity to a Japanese SAV strain (AB630068). The GIV strain shared 98.1% sequence identity with an SAV isolate from the USA (MG012424). The remaining GV SAV genome sequences were 98.5% identical to another Japanese strain (AB775659) isolated from a foodborne gastroenteritis outbreak [10]. The GIV and GV sequences from this study were the first complete genome sequences of members of these genotypes obtained in China.

To investigate the prevalence of SAVs in the 140 individual fecal samples, universal primers capable of detecting human SVA genogroups I, II, IV and V as well as genogroup-specific primers were used for conventional PCR screening [11]. showed that 19% (19/100) Sanger sequencing of 420-bp PCR products of the screening assay of the samples from diarrheic children were positive for SAVs, 12 of which belonged to GI, two of which belonged to GII, three of which belonged to GIV, and two of which belonged to GV. None of the 40 samples from healthy children were positive for any of the four genogroups of SAV. The prevalence results were in accordance with the results from metagenomics.

In summary, we detected SAVs belonging to genotypes GI, GII, GIV and GV in fecal samples from children with AGE in China. Eight complete SAV genome sequences were determined, including the first SAV GIV and GV genome sequences reported in China. A prevalence investigation indicated that 19% of the children with AGE of unknown etiology were positive for SAV. Our data suggest that SAVs are important pathogens associated with AGE in children in Jiangsu, China.

## Data Availability

The 14 sets of raw sequence reads were deposited in the Short Read Archive of the GenBank database with accession nos. SRX6651218-SRX6651231. The complete genome sequences of SAVs determined in this study were submitted to GenBank with the accession nos. MN161590- MN161597.

## References

[1] Svraka S, Vennema H, van der Veer B (2010). Epidemiology and genotype analysis of emerging sapovirus-associated infections across Europe. J Clin Microbiol.

[2] Lee LE, Cebelinski EA, Fuller C (2012). Sapovirus outbreaks in long-term care facilities, Oregon and Minnesota, USA, 2002–2009. Emerg Infect Dis.

[3] Kobayashi S, Fujiwara N, Yasui Y (2012). A foodborne outbreak of sapovirus linked to catered box lunches in Japan. Arch Virol.

[4] Magwalivha M, Kabue J-P, Traore AN, Potgieter N (2018). Prevalence of human sapovirus in low and middle income countries. Adv Virol.

[5] Diez-Valcarce M, Castro CJ, Marine RL (2018). Genetic diversity of human sapovirus across the Americas. J Clin Virol.

[6] Oka T, Wang Q, Katayama K, Saif LJ (2015). Comprehensive review of human sapoviruses. Clin Microbiol Rev.

[7] Zhang W, Li L, Deng X (2016). Viral nucleic acids in human plasma pools. Transfusion.

[8] Deng X, Naccache SN, Ng T (2015). An ensemble strategy that significantly improves de novo assembly of microbial genomes from metagenomic next-generation sequencing data. Nucleic Acids Res.

[9] Zhang W, Yang S, Shan T (2017). Virome comparisons in wild-diseased and healthy captive giant pandas. Microbiome.

[10] Shibata S, Sekizuka T, Kodaira A, et al (2015) Complete Genome Sequence of a Novel GV.2 Sapovirus Strain, NGY-1, Detected from a Suspected Foodborne Gastroenteritis Outbreak. Genome Announc 3. 10.1128/genomeA.01553–14 10.1128/genomeA.01553-14 PMC4333662 25676762

[11] Okada M, Yamashita Y, Oseto M, Shinozaki K (2006). The detection of human sapoviruses with universal and genogroup-specific primers. Arch Virol.

